# Diversity profiling of soil bacterial and fungal communities in the Ogasawara (Bonin) Islands, Japan

**DOI:** 10.1128/MRA.00644-23

**Published:** 2023-09-21

**Authors:** Nobuhiko Shigyo, Koji Shichi, Kyoko Sugai, Suzuki Setsuko

**Affiliations:** 1 Department of Forest Soils, Forestry and Forest Products Research Institute, Ibaraki, Japan; 2 Shikoku Research Center, Forestry and Forest Products Research Institute, Kochi, Japan; 3 Institute of Agricultural and Life Sciences, Academic Assembly, Shimane University, Shimane, Japan; 4 Department of Forest Molecular Genetics and Biotechnology, Forestry and Forest Products Research Institute, Ibaraki, Japan; California State University San Marcos, San Marcos, California, USA

**Keywords:** amplicon sequencing, soil bacteria, soil fungi, oceanic islands

## Abstract

Island biogeography research provides insight into microbial diversity patterns; however, little is known about the diversity and distribution of soil microbial communities on remote and poorly accessible islands. Here, we present amplicon sequencing data from bacterial and fungal communities in the surface soils of the Ogasawara (Bonin) Islands, Japan.

## ANNOUNCEMENT

Studies of island biogeography provide valuable insight into the underlying patterns that govern microbial diversity (1, 2). However, there is a lack of information on the diversity and distribution of soil microbes on remote and inaccessible islands. The Ogasawara Islands, which are located in the northwestern Pacific Ocean and isolated from any mainland, represent a unique and vulnerable ecosystem (3). Here, we provide a report of soil bacterial and fungal communities on the Ogasawara (Bonin) Islands based on amplicon sequencing. The data provided here will facilitate future studies of biogeography and conservation on oceanic islands.

Sampling was conducted on two of the Ogasawara Islands (Chichijima and Hahajima Islands; Fig. 1A) in November 2020. Soil samples were collected from the O-horizon and the top 10 cm of the mineral horizon at two sites on Chichijima Island, YAK1 (27°05.6014′ N, 142°13.0323′ E) and YAK2 (27°05.6172′ N, 142°13.0367′ E), and one site on Hahajima Island, HHI1 (26°37.1788′ N, 142°10.7763′ E). These sites were located in subtropical forests mainly composed of *Pandanus boninensis* and *Livistona boninensis*. Each sample was placed in a sterile sampling bag, and then kept in a cooler with ice until transported to the laboratory, where it was stored at −80°C until downstream analysis. Soil DNA was extracted using the NucleoSpin Soil DNA extraction kit (Macherey-Nagel, Düren, Germany), after which amplicon libraries were prepared by a two-step tailed PCR procedure as described in our previous studies (4, 5). The 515F/806R primer pair [5′-GTGCCAGCMGCCGCGGTAA-3′/5′-GGACTACHVGGGTWTCTAAT-3′; (6)] was chosen to target the V4 hypervariable region of the prokaryotic 16S rRNA gene. For fungal communities, the gITS7/ITS4ngs primer pair [5′-GTGARTCATCGARTCTTTG-3′/5′-TTCCTSCGCTTATTGATATGC-3′; (7)] was used to target the ITS2 region. Sequencing of bacterial and fungal communities was performed on the Illumina MiSeq platform using 2 × 250 bp and 2 × 300 bp paired-end reads, respectively (FASMAC Co., Ltd., Kanagawa, Japan) (Table 1). The raw reads were analyzed using the DADA2 pipeline, version 1.26.0 (8). Bacterial sequences followed the DADA2 tutorial (https://benjjneb.github.io/dada2/tutorial.html) with default parameters, except for truncLen = c(240,210). Fungal sequences were processed using the DADA2 ITS workflow (https://benjjneb.github.io/dada2/ITS_workflow.html). Taxonomic assignments for bacteria were made using the SILVA database, version 138.1 (9), subsequently excluding archaeal, mitochondrial, and chloroplast sequences. Fungal taxonomic affiliations were based on the UNITE database, version 9.0 (10). Bacterial and fungal sequences from each sample were randomly rarefied to 32,222 and 23,124 sequences, respectively, based on the sample with the lowest number of sequencing reads.

**Fig 1:**
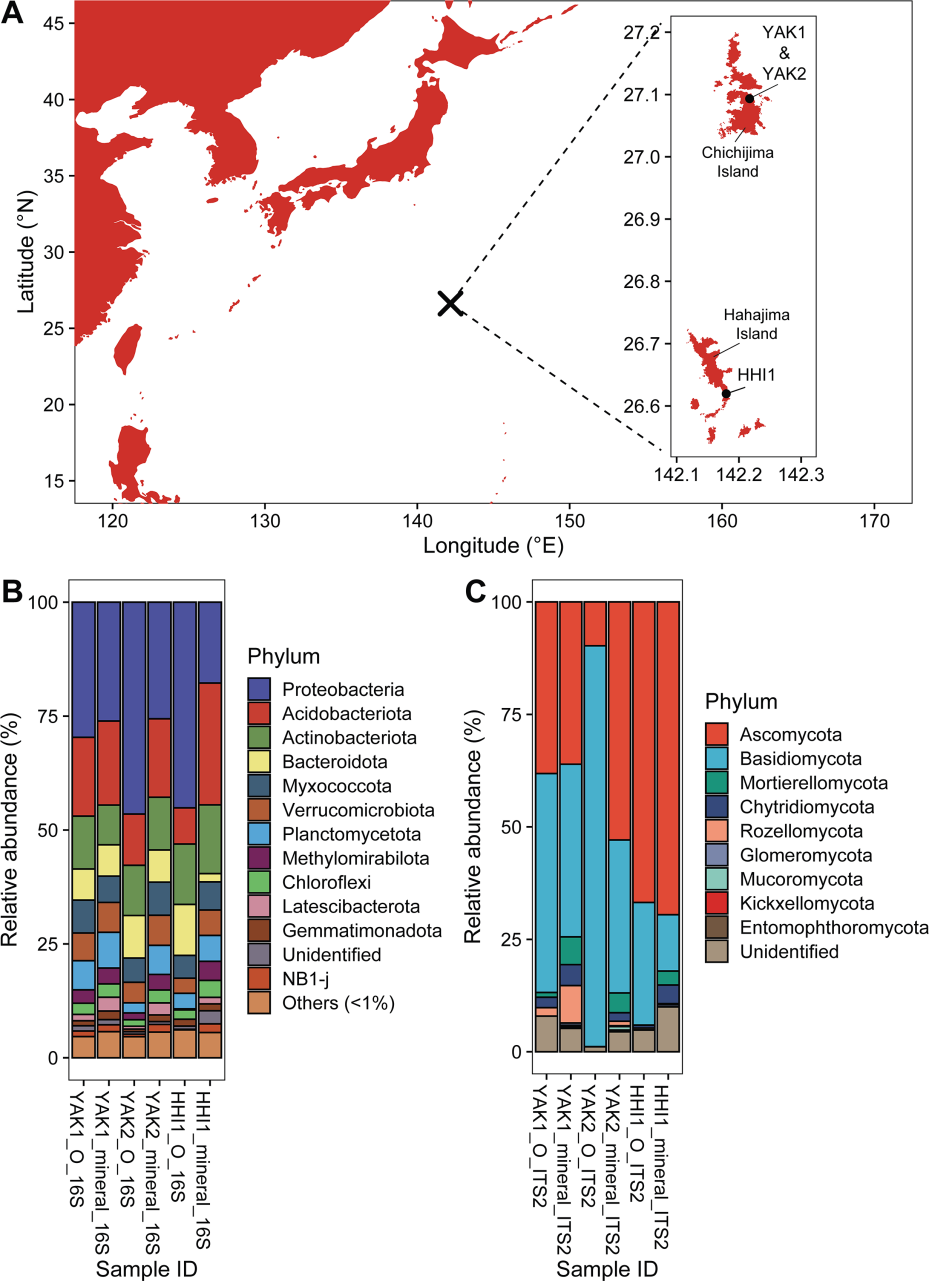
(**A**) Location of sampling sites within the Ogasawara (Bonin) Islands, in the northwestern Pacific Ocean. O-horizon and surface mineral soil samples were collected from three sites: YAK1, YAK2, and HHI1. (**B**) Relative abundance of soil bacterial communities at the phylum level. (**C**) Relative abundance of soil fungal communities at the phylum level.

**TABLE 1:** Summary of demultiplexed sequencing data

Sample ID	Site ID	Islands	Soil horizons	No. of raw reads	BioSample accession no.	SRA accession no.
YAK1_O_16S	YAK1	Chichijima Island	O-horizon	290,326	SAMD00579159	DRR483123
YAK1_mineral_16S	YAK1	Chichijima Island	Mineral horizon	88,505	SAMD00579160	DRR483124
YAK2_O_16S	YAK2	Chichijima Island	O-horizon	285,573	SAMD00579161	DRR483125
YAK2_mineral_16S	YAK2	Chichijima Island	Mineral horizon	95,422	SAMD00579162	DRR483126
HHI1_O_16S	HHI1	Hahajima Island	O-horizon	348,189	SAMD00579163	DRR483127
HHI1_minetal_16S	HHI1	Hahajima Island	Mineral horizon	92,273	SAMD00579164	DRR483128
YAK1_O_ITS2	YAK1	Chichijima Island	O-horizon	105,315	SAMD00579165	DRR483129
YAK1_mineral_ITS2	YAK1	Chichijima Island	Mineral horizon	123,264	SAMD00579166	DRR483130
YAK2_O_ITS2	YAK2	Chichijima Island	O-horizon	93,339	SAMD00579167	DRR483131
YAK2_mineral_ITS2	YAK2	Chichijima Island	Mineral horizon	123,569	SAMD00579168	DRR483132
HHI1_O_ITS2	HHI1	Hahajima Island	O-horizon	131,532	SAMD00579169	DRR483133
HHI1_mineral_ITS2	HHI1	Hahajima Island	Mineral horizon	114,818	SAMD00579170	DRR483134

The prevailing bacterial phyla were Proteobacteria (O-horizon: 29.3–46.4%, mineral horizon: 17.4–26.0%), Acidobacteriota (O-horizon: 8.0–17.4%, mineral horizon: 17.5–27.0%), Actinobacteriota (O-horizon: 11.1–13.6%, mineral horizon: 8.9–14.9%), and Bacteroidota (O-horizon: 6.8–11.1%, mineral horizon: 1.8–6.9%; Fig. 1B). The dominant fungal phyla comprised Ascomycota (O-horizon: 9.8–66.4%, mineral horizon: 36.0–69.4%), Basidomycota (O-horizon: 27.3–88.9%, mineral horizon: 12.6–38.3%), Mortierellomycota (O-horizon: 0–1.1%, mineral horizon: 3.1–6.1%), and Chytridiomycota (O-horizon: 0–2.3%, mineral horizon: 1.9–4.8%; Fig. 1C).

## Data Availability

The amplicon data are available in the DNA Data Bank of Japan Sequence Read Archive under accession number DRA016470.
